# Reactive attachment disorder symptoms and prosocial behavior in middle childhood: the role of Secure Base Script knowledge

**DOI:** 10.1186/s12888-020-02931-3

**Published:** 2020-11-04

**Authors:** Bien Cuyvers, Eleonora Vervoort, Guy Bosmans

**Affiliations:** 1 Department of Clinical Psychology, Katholieke Universiteit Leuven, Tiensestraat 102, 3000 Leuven, Belgium; 2 Leuven, Belgium

**Keywords:** Reactive attachment disorder symptoms, Prosocial behavior, Secure base script

## Abstract

**Background:**

Children with attachment disorder show prosocial behavior problems. Children with a reactive attachment disorder show inhibited and emotionally withdrawn behavior. Consequently, these children typically display prosocial behavior problems. However, the underlying mechanism between reactive attachment disorder and prosocial behavior problems is still unclear and findings in literature are mixed.

**Methods:**

The current study investigated the role of children’s attachment representations in this association. Attachment representations reflect knowledge about a cognitive script regarding the attachment figure as a source for support (Secure Base Script). We tested whether secure base script knowledge 1) mediates or 2) moderates the link between reactive attachment disorder and prosocial behavior problems in 83 children (6–11 years; 83.1% boys) recruited from special education schools for children with behavioral problems. Children completed a pictorial Secure Base Script Test. Their reactive attachment disorder symptoms were assessed during an interview with the primary caregivers. Primary caregivers and teachers filled out a prosocial behavior questionnaire about the child.

**Results:**

Results did not support the mediation hypothesis, but evidence for the moderation hypothesis was found. Secure base script knowledge attenuated the negative association between attachment disorder symptoms and prosocial behavior.

**Conclusions:**

These findings contribute to the discussion about the link between attachment representations and attachment disorders.

**Supplementary Information:**

The online version contains supplementary material available at 10.1186/s12888-020-02931-3.

## Background

Children who develop reactive attachment disorder (RAD) symptoms show inhibited, emotionally withdrawn behavior towards adult caregivers, and/or persistent social or emotional disturbances, reflected in minimal social and emotional responsiveness, limited positive affect or episodes of unexplained arousal [1]*.* As part of this symptomatology, these children typically show prosocial behavior problems [2, 3]. Their inability to relate prosocially to and establish deep bonds with peers and adult caregivers adds significantly to the distress of these children and their environment [4, 5] and puts these children at risk of not having the appropriate resources to receive help or support when needed [6]. Further, these children are at elevated risk to develop other symptoms of psychopathology later in life (e.g. externalizing problems) [7]. So far, little is known about *why* these children show impaired prosocial behavior. Nonetheless, a better understanding of this association could not only inform theory about RAD symptoms, but also inform clinical practice in order to better support these children. In the current study, we focused on the role of attachment representations and tested whether these representations either mediate or moderate the link between RAD symptoms and prosocial behavior problems.

Research shows that the severity of RAD symptoms is, among other behavior, reflected in the extent to which these children display prosocial behavior problems during interactions with peers [8, 9]. Prosocial behavior can be defined as behavior that is meant to benefit others rather than oneself [10] and results from a process consisting of three subsequent steps reflecting 1) the ability to take perspective, 2) the ability to determine the cause of the problem, and 3) the wish to help the other [11]. This process can result in different prosocial behavior types, such as helping (responding to instrumental needs), sharing (responding to unmet material desire) and comforting (responding to unmet emotional desire) [11]. The importance of prosocial behavior cannot be underestimated as a precursor for adaptive behavior throughout the lifespan. Research found that prosocial behavior results in more positive affect later in life [12], more quality friendships [13], less delinquency [14], and higher self-esteem and subjective well-being [15]. Stimulating, and therefore understanding, the development of prosocial behavior is important [16]. Especially for children with RAD symptoms, who already have difficulties bonding and forming meaningful relationships, a good development of prosocial behavior is of high importance [8]. However, little is known about the factors that explain the link between RAD symptoms and decreased prosocial behavior. Because RAD symptoms are assumed to be linked to prosocial behavior problems due to underlying problems in emotion regulation [17], and because emotion regulation is highly affected by children’s attachment development [18], the current study focuses on the role of attachment in the association between RAD symptoms and prosocial behavior problems. Thus far, how these factors relate to each other has been little studied. Therefore, the current study aims to test two hypotheses.

The first hypothesis proposes that Internal Working Models about attachment [19] mediate the link between RAD symptoms and prosocial behavior problems in middle childhood. Internal Working Models are mental representations about the care-related interactions with the caregiver [20]. Waters and Waters [21] later demonstrated that secure Internal Working Models consist at least partly of a Secure Base Script (SBS). A SBS is a causally linked chain of expected events that starts with the expectation that distress is followed by support seeking, which activates caregiver comfort, support and help. This results in a reduction of distress and ends with a sense of being back on track [20]. The level of knowledge about such a SBS mirrors the extent to which children are less or more securely attached [22].

**Fig. 1:**
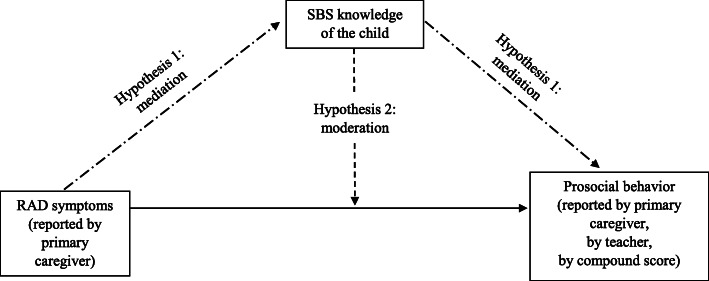
Schematic presentation of the two hypotheses in the current study

The current mediation hypothesis follows psychiatric theory that assumes a unique link between RAD symptoms and insecure attachment [23]. The attachment disorder terminology seems to suggest that insecure attachment representations, (i.c., lack of SBS knowledge) are the core and most specific issue when children show RAD symptoms. For example, it has been found that children of parents that expressed characteristics interfering with a normal attachment, such as depression, lack of social support and isolation, process showed more severe RAD symptoms [24, 25]. Further, Van Ijzendoorn and Bakermans-Kranenburg suggest that RAD symptoms can be linked to attachment disturbances for children experiencing frightening parental behavior [26]. In all, theory predicts to find that all children with RAD symptoms display ruptured trust in the availability of their primary caregivers, mirrored in insecure attachment representations. In turn, attachment theory assumes that insecure attachment representations are linked with children’s decreased prosocial behavior [27]. Theory suggests that decreased prosocial behavior serves a protective function for insecurely attached children: because they anticipate that they cannot rely on others for support they are more motivated to create social distance to avoid further relational pain [28, 29]. In sum, one would expect children with more RAD symptoms to have more insecure attachment representations (i.c., less SBS knowledge), therefore, one could expect that SBS mediates the link between RAD symptoms and reduced prosocial behavior (see Fig. 1).

Although this mediation hypothesis aligns with ruling theory, research shows that it is surprisingly difficult to find evidence in favor of a robust association between measures of both constructs of RAD symptoms and attachment security [30–32]. For example, recent research of Schröder and colleagues showed that insecure attachment representations and RAD symptoms are distinct concepts [33]. They found no significant differences in the occurrence of the different attachment representations (secure, avoidant, anxious and disorganized) in children in early middle childhood with RAD symptoms. In the same vein, research of Minnis and colleagues found that some children with RAD symptoms still show secure attachment to their caregivers which contradicts the idea that insecure attachment is the core and most specific issue when children show RAD symptoms [34]. These and similar studies led some attachment disorders researchers to formulate alternative theories on why children develop RAD symptoms.

For example, Green stated that RAD symptoms are represented by social behavior problems rather than attachment related problems [35]. Also, Rutter, Kreppner, and Sonuga-Barke reviewed empirical research on attachment disorders and concluded that RAD symptoms are indicative of emotional and behavioral dysregulation in general instead of attachment insecurity specifically [36]. In a similar vein, other scholars argued that RAD symptoms might reflect not so much individual differences in attachment (in)security but reflect hyperarousal linked to post traumatic stress disorder or similar anxiety-related disorders [37–39]. Research further suggests that RAD symptoms and attachment have a different etiological background. For example, RAD symptoms typically develop in the context of aversive early life events such as severe early deprivation [40], while such events have a less robust effect on attachment development [41, 42]. Hence, it remains a theoretical and empirical question whether RAD symptoms necessarily reflect insecure attachment. This raises the question whether attachment representations could play a different role than mediator in the link between RAD symptoms and prosocial behavior problems.

The second hypothesis proposes that attachment representations may play a moderating role in the link between RAD symptoms and prosocial behavior problems. Accumulating research shows that secure attachment buffers against the maladaptive effects of stress on the severity of psychopathology symptoms. For example, Dujardin et al. showed that stress leads to less depressive symptoms in more securely attached children because they are better able to seek support [43]. Similarly, attachment moderates the effect of prenatal stress on later child fearfulness [44], and individuals vulnerable to develop psychosis have less severe psychotic symptoms when distressed if they are more securely attached [45]. In all, several studies have shown that individual differences in attachment determine the severity of the symptoms associated with specific psychiatric disorders [46, 47]. In line with these studies, the current study aimed to test the hypothesis that children high on RAD symptoms with more SBS knowledge show less decreased prosocial behavior than children high on RAD symptoms with less SBS knowledge (see Fig. 1).

## Methods

### Current study

The current study will test the hypotheses that the link between RAD symptoms and prosocial behavior problems is (a) mediated, or (b) moderated by SBS knowledge. We collected data in children in early middle childhood, an age characterized with increasing social cognitive script-like learning [48]. This suggests that this age is appropriate to study the role of SBS knowledge in children with RAD symptoms. To investigate our research questions, we collected data in a sample of children with special educational needs due to emotional and behavioral problems (in Belgium: type 3, special education). Conducting the study in this population increased the likelihood of identifying a relevant number of children with RAD symptoms. Moreover, studying this population allowed us to evaluate the extent to which less SBS knowledge is a problem that is specific for children with RAD symptoms compared to children with other emotional and behavioral problems. We controlled for gender and age because previous studies encountered gender and age differences in prosocial behavior [49, 50].

### Participants

For this study, a sample of school children with special educational needs due to severe behavioral problems was recruited. First, we contacted 38 special educational schools in Flanders of which 21 schools agreed to participate. Of the 425 people contacted for this study, the primary caregivers of 116 children (27%) born between 2001 and 2004 signed the informed consent and 83 (20%) of them conducted the interview about RAD symptoms in their child (Disturbances of Attachment Interview, DAI) [51]. There were 22 children who reached the threshold for RAD symptoms.

Maximally two children per teacher could participate in this study to avoid influence of possible bias about prosocial behavior of the teacher. The children’s ages ranged between 6.22 and 10.39 years old (*M* = 8.35, *SD* = 0.96). Most of them (98,8%) were Caucasian. Of these 83 children, 83.1% were boys. For 48% of the children, the school psychologists reported a history of pathogenic care (e.g. maltreatment, neglect, or abuse). Furthermore, children staying at a special boarding school with multiple caregivers during the week but not the weekend, accounted for 39% of the sample. 6.8% of primary caregivers had a university degree, 12.4% had a higher education degree, 43.8% had a secondary school degree, and 27.0% had a primary school degree. This study was approved by the KU Leuven’s ethical committee (SMEC).

### Materials and procedure

#### Attachment disorder symptoms

To measure RAD symptoms, the Disturbances of Attachment Interview (DAI) [51] was administered by children’s primary caregivers. The DAI is a semi-structured interview, initially meant for children between 1 and 5 years of age. Later, Smyke and Zeanah adapted the interview for use with children in middle childhood, containing 11 questions about RAD symptoms. Scores on each question ranged between 0 and 2 (0 being ‘*behavior not present’*, 1 being ‘*behavior present to some extent’*, 2 being ‘*behavior consistent with attachment disorder’*), with a total score between 0 and 10. Smyke, Dumitrescu, and Zeanah divided the scores on the DAI into four categories, with 1) children with no signs of RAD symptoms, 2) children with no selective attachment, moderate indiscriminate and inhibited but somewhat emotionally responsive behavior, 3) children with a selective attachment and high levels of indiscriminate behavior, and 4) children with no selective attachment and high levels of both inhibited and indiscriminate behavior [52].

We conducted Principal Component Analyses with varimax rotation on the scores and found two components, reflecting inhibited (4 items) and disinhibited behavior (7 items). We only focused on the Inhibited scale in the current research because according to the novel DSM-5 guidelines, only the Inhibited scale refers to attachment disorder symptoms. Gleason et al. found an association between the Inhibited scale of the DAI and attachment behavior in children with a mean age of 22 months [53]. Further, they found the DAI to reach convergence with a psychiatric diagnostic interview, and show discriminant validity with depression. Also, Giltaij, Sterkenburg, and Schuengel found that results of the DAI are strongly correlated to clinically observed attachment-disorder related behavior [54]. Interrater reliability and internal consistency substantiated to be acceptable for the inhibited component [52] and these were valid in this study as well. Spearman Rho correlation based on 22 (28,6%) double-coded interviews was .89. In contrast, Cronbach’s alpha for this component was low (.53), so it could have influenced the results. Therefore, we calculated a factor score and redid the analyses. The pattern of effects did not change substantially, which indicates that error variance did not affect the results. We worked with the sum-score of RAD symptoms for each child separately.

#### Secure Base script knowledge

**Fig. 2:**
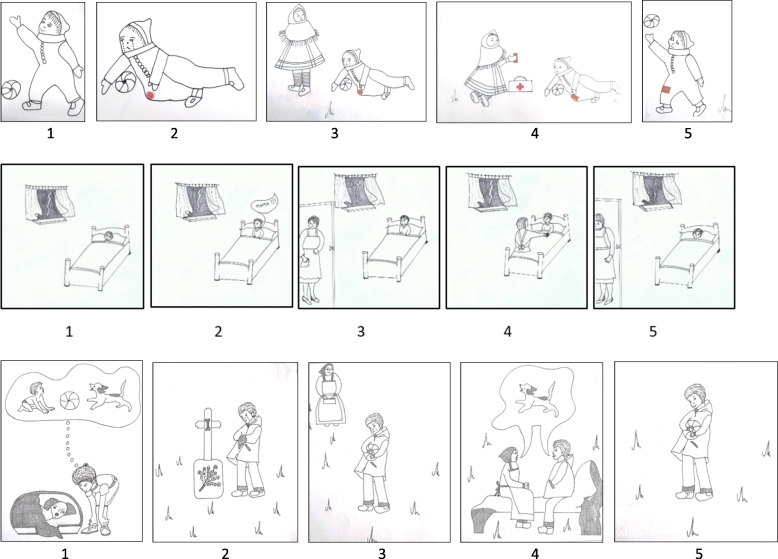
Pictures in correct order of the Secure Base Script pictorial test, developed by Bosmans, Spilt, Vervoort, Verschueren

To assess SBS knowledge, research typically uses prompt words [21]. However, we were concerned that the current sample of children with special educational needs at this young age would be delayed in their narrative capacities decreasing the validity of the test [55, 56]. Based on Waters and Waters’ idea that children with more SBS knowledge recognize SBS information faster than children with less SBS knowledge [21], we developed a non-verbal version of the SBS assessment task (Bosmans, Spilt, Vervoort, Verschueren). SBS knowledge was measured as speed of recognition of the SBS in a non-verbal Secure Base Script pictorial test (see Fig. 2). In this test, five pictures were offered in the same wrong order for all children. Children were asked to organize pictures in such a way that they followed a logical story line (see Fig. 2): “I here have different cards with different pictures on them. These cards can form a story together, but I did not put the cards in the right order. The aim is to put these pictures in an order so that the story makes sense. We will start with the first story. The first two cards are already put in the correct order. On the first picture, you see a child playing. On the second card you can see that the child has fallen down. Can you put the other three cards in the right order so that the story makes sense? Work as fast as you can and tell me when you are done”. For the second story, the instructions: “This is the second story. The first two cards are again already put in the correct order. On the first card you can see a child lying in bed. It is storming outside. On the second card you can see that the child is scared of the storm and calls its mom. Can you put the other three cards in the right order so that the story makes sense? Work as fast as you can and tell me when you are done”. Then the last story follows with the instructions: “This is the third story. Again, the first two cards are already in the correct order. On the first picture, you can see a child thinking how he used to play with his old dog when he was younger. On the second picture, you can see that the dog has passed away and is buried outside. The child is crying at his grave. Can you put the other three cards in the right order so that the story makes sense? Work as fast as you can and tell me when you are done”. The story line represented a SBS related set of events in which the mothers perceives the child’s need for help, she then provides help or support, after which the child feels back on track. We measured how much time children needed to solve three SBS stories containing five pictures each and the children’s accuracy solving the task. The theory of SBS knowledge refers to the speed of accurately recognizing SBS content. We were concerned that just relying on the accuracy scores would induce variance unrelated to attachment in the data due to the fact that children could either accidently, or after trial and error without actual insight put the items in the correct order. Therefore, we used both indicators of accuracy and reaction time to calculate a continuous score reflecting the time they needed to solve a SBS story correctly.^1^ Thus, for all correctly solved stories, the mean reaction time was measured. Hence, higher reaction times are indicated by lower scores and thus less SBS knowledge, while lower reaction times are indicated by higher scores and thus more SBS knowledge. For the first story, 13 children failed to solve the stories correctly, for the second story almost half of the children failed (*n* = 40) to put the pictures in the correct order, and for the third story, even more children did not succeed to put the pictures correctly (*n* = 60). Mean reaction times for the first, second and third story were 11.5 s, 7.9 s and 6.0 s respectively. The scores on the first story were normally distributed, but the scores on the second and third story were concentrated around zero. Correlations between the stories ranged between .18 (*p* = .107) and .42 (*p* < .001) and Cronbach’s α was low (α = .50). This pointed at low internal consistency, possibly due to the low number of items, namely three [57]. To investigate whether it was statistically warranted to aggregate across the items, we explored the eigenvalues and found that only one eigenvalue reached a value above 1 (eigenvalue = 1.56). This suggests there is only one underlying factor in these three items. The first item explained 51.83% (factor loading = 0.76), the second explained 28.80% (factor loading = .80) and the third item explained 19.37% (factor loading = .51) of the variance in this pictorial Secure Base Script knowledge test. All three items thus reach factor loadings above .40, which is argued as acceptable for measures with such a small number of items [58].

#### Prosocial behavior

A subscale of the Strengths and Difficulties Questionnaire (SDQ) allowed us to assess prosocial behavior, reported by children’s primary caregivers and their teacher. The questionnaire was developed for reporting psychosocial problems by primary caregivers and teacher about children between 2 and 17 years old. Questions asked about the behavior of children in the past six months (e.g., *My child is considerate of other people’s feelings)*. Every item was rated on a three point Likert scale as ‘*Not true’*, ‘*Somewhat true’* or ‘*Certainly true’*. These answers were coded into 0, 1 or 2, respectively. The Prosocial Behavior subscale consisted of the sum of scores on 5 items (α = .72 for primary caregivers, α = .73 for teachers), ranging from 0 to 10. Higher scores imply more prosocial behavior while lower scores indicate less prosocial behavior. In addition, we calculated a primary caregiver-teacher reported compound of the prosocial behavior score by summing the mean of the standardized values of primary caregiver and teacher reports of prosocial behavior.^2^

#### Perceptual intelligence

Because intelligence plays a role in the reaction time in pictorial tests, we controlled for perceptual intelligence in all the analyses in which we included data from the SBS knowledge pictorial test. Three items of a subtest of the Wechsler Intelligence Scale for Children-Third Edition in Dutch (WISC-III NL) [59], namely ‘Picture Arrangement’, were completed by all children. The test measures perceptual intelligence in children between 6 and 17 years old. It assesses a child’s capacity to interpret pictorial social cues and to organize them in a logical sequence. In this task, children are presented with a set of pictures in the wrong order. The aim is for children to rearrange the pictures in the right order so they tell a logical story. All three items were scores as either 1, meaning the child managed to put the pictures in the correct order, or 0 when the item was solved incorrectly. Also, for every item the time needed to provide a solution was noted, in seconds. Then, for all correctly solved items, a mean reaction time score was calculated for each child (α = .54). Last, to increase the interpretability we reversed all scores so lower values reflected less perceptual intelligence and higher scores reflected better perceptual intelligence.

## Results

### Preliminary analyses

As shown in Table 1, RAD symptoms were linked to less prosocial behavior according to the primary caregiver and the primary caregiver-teacher compound reports. Prosocial behavior was not significantly correlated over informants (*r* = .18, *p* = .097). This finding is in line with other (SDQ) studies showing little convergence between caregiver and teacher reports of children’s social problem [60]. Age was positively correlated with SBS knowledge. This suggests that, with increasing age, children displayed more SBS knowledge, which is expected based on previous SBS research in middle childhood [21]. In addition, perceptual intelligence was positively correlated with SBS knowledge suggesting that part of the variance in our measure of SBS knowledge related to intelligence. Primary caregiver’s education level correlated with prosocial behavior reported by primary caregivers and the compound score of caregiver and teacher reports. Therefore, we also repeated the analyses predicting caregiver-reported prosocial behavior including primary caregiver’s education level as an additional covariate.

**Table 1:** Means, Standard Deviations, and Correlations between The Study’s Main Variables, Age, Gender, perceptual intelligence and caregiver’s educational level

	1	2	3	4	5	6	7	8	9
1. RAD symptoms	–								
2. SBS knowledge	−.12	–							
Prosocial behavior									
3. by primary caregiver	−.30^**^	.17	–						
4. by teacher	−.12	.07	.18	–					
5. compound score	−.28^*^	.14	.77^**^	.77^**^	–				
Demographic variables									
6. Age	−.03	.27^*^	−.00	−.06	−.03	–			
7. Gender	−.08	.00	−.02	−.08	−.06	.04	–		
8. Perceptual intelligence	.04	.55^**^	.08	.01	.06	.26	.17	–	
9. Caregiver’s educational level	.10	.13	−.23	−.29*	−.34**	−.06	.25	.07	–
*Mean*	.52	−16.35	6.09	5.47	0	8.75	1	15.24	2.89
*Standard Error*	.31	7.15	2.49	2.61	1	.97	0	6.85	1.27
*N*	83	85	82	83	82	83	83	82	75

*Note*. *** *p* < .001; ** *p* < .01; * *p* < .05; Gender is dummy coded (girl = 0, boy = 1), caregiver’s educational level is coded into 6 categories

### Hypothesis 1: testing the mediation models

Table 1 shows that there was no significant correlation between RAD symptoms and performance on the SBS task when we controlled for age, gender, perceptual intelligence (WISC-III-NL). Even when we controlled for caregiver’s educational level the effect remained non-significant. This already contradicts the mediation hypothesis. Nevertheless, we conducted the mediation analyses as planned. For this purpose, we used the PROCESS macro in SPSS [61], with RAD symptoms as the predictor, SBS knowledge as the mediator and prosocial behavior as the dependent variable. Analyses were conducted with 5000 bootstraps. Participants with missing data in any of the variables were removed from the analyses in PROCESS.

In line with what could be expected from the correlation analyses, we found no significant mediation effects of SBS knowledge in the link between RAD symptoms and prosocial behavior, reported by primary caregivers (β = − 0.22, 90% CI [− 0.72, 0.15], see Fig. S1a), by teacher (β = − 0.18, 90% CI [− 0.71, 0.19], see Fig. S1b), nor by the compound scores of both informants (β = − 0.07, 90% CI [− 0.23, 0.04], see Fig. S1c). Because in each of these analyses, zero is part of the confidence interval, we found no support for our first hypothesis that SBS knowledge is a mediator in the link between RAD symptoms and prosocial behavior problems. Only the direct effects between RAD symptoms and prosocial behavior reported by the primary caregiver and the mean standardized score of both informants was significant (see Fig. S1a and Fig. S1c). Consequently, we found no support for our first hypothesis that SBS knowledge would be a mediator in the link between RAD symptoms and prosocial behavior problems.

### Hypothesis 2: testing the moderation models

To investigate our second hypothesis, we conducted three moderation analyses with the PROCESS macro of SPSS [61]. We thereby took RAD symptoms as the predictor, SBS knowledge as the moderator and prosocial behavior (primary caregiver report, teacher report, and compound report) as outcome variables. Analyses were conducted with 5000 bootstraps. Participants with missing data in any of the variables were removed from the analyses in PROCESS.

**Fig. 3:**
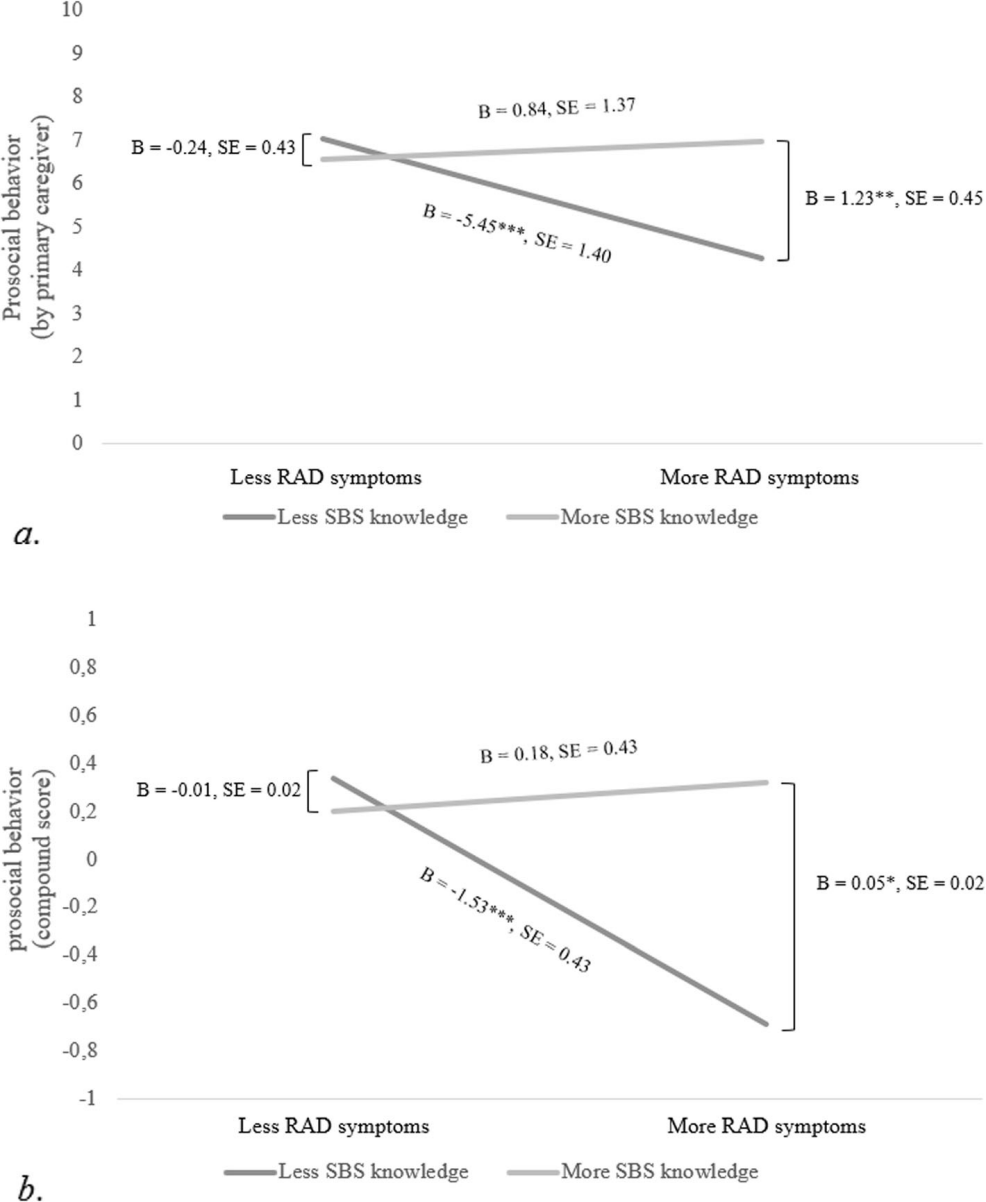
**a**. Interaction effect controlled for age, gender and intelligence (WISC-III-NL), with Prosocial behavior evaluated by primary caregivers as dependent variable. *p < .05, **p < .01, ***p < .001. **b**. Interaction effect controlled for age, gender and intelligence (WISC-III-NL), with the mean standardized scores of prosocial behavior evaluated by primary caregivers and teacher as dependent variable (compound score). *p < .05, **p < .01, ***p < .001

Table 2 shows the detailed results of the moderation analyses. When prosocial behavior was reported by primary caregivers, the interaction between RAD symptoms and SBS knowledge was significant. Controlling for age, gender, perceptual intelligence and caregiver’s educational level did not affect the interaction effect. Figure 3a shows that the association between RAD symptoms and prosocial behavior was only significant for children with less SBS knowledge. For these children, more RAD symptoms linked to less prosocial behavior. For children with more SBS knowledge, there was no association between RAD symptoms and prosocial behavior. Further probing of the interaction effect showed that for children with less RAD symptoms, there was no association between SBS knowledge and prosocial behavior. Instead, for children with more RAD symptoms, more SBS knowledge was linked with more prosocial behavior. When prosocial behavior was reported by teachers, the interaction between RAD symptoms and SBS knowledge was not significant (see Table 2). When prosocial behavior was derived from the primary caregivers-teacher compound score, similar significant interaction effects between RAD symptoms and SBS knowledge were found as described above (see Fig. 3b). This effect remained significant after controlling for age, gender, perceptual intelligence and caregiver’s educational level. This thus suggests that SBS knowledge buffers against the severity of prosocial behavior problems in children with RAD symptoms.

**Table 2:** Linear Regression of the Interaction between RAD symptoms and SBS knowledge on Prosocial Behavior reported by primary caregiver, by teacher, and their mean standardized compound score

	Prosocial behavior by primary caregiver		Prosocial behavior by teacher		Prosocial behavior compound score	
	*β*	*∆R*^*2*^ *(f*^*2*^*)*	*β*	*∆R*^*2*^ *(f*^*2*^*)*	*β*	*∆R*^*2*^ *(f*^*2*^*)*
*Step 1*		.121 (.138)		.048 (.050)		.122 (.139)
RAD symptoms	−.316**		−.127		−.299**	
SBS knowledge	.198		.158		.203	
Age	−.122		−.107		−.134	
Gender	−.091		−.125		−.140	
Perceptual intelligence	−.018		−.039		−.025	
*Step 2*		.088 (.264)**		010 (.062)		.070 (.779)*
RAD symptoms x SBS knowledge	.308**		.102		.275*	

*Note.* *p < .05, **p < .01, ***p < .001; reported *β*’s reflect values at Step 2

## Discussion

The current study aimed to investigate a) whether lack of SBS knowledge mediates the association between RAD symptoms and prosocial behavior problems, and b) whether SBS knowledge moderates the association between RAD symptoms and prosocial behavior problems, in a population of children with special educational needs due to emotional and behavioral problems. Although, we did not find support for our first hypothesis, we found some support for the hypothesis that children with more RAD symptoms displayed more prosocial behavior if they had more SBS knowledge. In line with our prediction, SBS knowledge decreased RAD symptoms children’s risk to display less prosocial behavior.

With regard to our first hypothesis, SBS knowledge did not mediate the link between RAD symptoms and prosocial behavior. It could be that mediation effects would have emerged had we separately studied the different steps in the prosocial behavior process or the different types of prosocial behavior [11]. Research points at perspective-taking [62, 63] and comforting [64] as aspects of the complex prosocial behavior construct that are most closely related to attachment. Future research should focus on these aspects of prosocial behavior before the mediation hypothesis can be fully abandoned. Additionally, the current results might be attributed to the pictorial SBS measure. This measure was novel and used for the first time in the current study. The measure diverged from the much better studied word-prompt SBS measure where children are asked to use a list of words that roughly suggest a secure base story in order to create narratives that can be coded for SBS knowledge [21, 65, 66]. However, when developing the current study, we were worried that this narrative approach was going to be too difficult given children with RAD symptoms’ expected limited cognitive and narrative skills [67]. We were concerned that this could create ceiling effects suppressing the investigated correlations. Hence, the current approach has promising advantages compared to the word prompt approach, but could not reveal any link between RAD symptoms and SBS knowledge. Before drawing final conclusions about this lack of association, it will be necessary to conduct more validation research on the SBS pictorial test. Further, mediation effects might be stronger in samples with older participants. The review of Gross et al. showed that links between attachment and prosocial behavior are hard to find in middle childhood, but become more apparent in adolescence [64]. This is in line with research showing that SBS knowledge is still significantly developing in middle childhood [22]. It could be that, at ages when the SBS further matured, links with prosocial behavior are more easily found. Also, the sample size might have been too small to reveal effects. Therefore, if the study would be repeated in middle childhood, it could be relevant to collect data in larger samples such that even small effects can be identified.

Finally, the current pattern of results might be affected by the clinical nature of our sample. Insecure attachment is a transdiagnostic risk factor involved in several psychiatric disorders [68]. Hence, one could argue that the lack of a correlation between RAD symptoms and SBS knowledge reflects that children with other psychiatric problems have similar attachment-related issues. Alternatively, we could have compared children with RAD symptoms to healthy controls. However, if such a comparison would have revealed a link between RAD symptoms and SBS knowledge, it would still have been impossible to claim that insecure attachment is the core and most specific issue that children with ASD struggle with, because theory suggests that the average child with any mental health problem will have less SBS knowledge than healthy controls [66]. This way, the current findings resonate with other studies that failed to find a link between RAD symptoms and insecure attachment [37] and with a mounting number of scholars argueing that insecure attachment is not the core and specific issue characterizing attachment disorder symptoms [27, 35, 36, 38].

With regard to our second hypothesis, we found some support that SBS knowledge functions as a moderator in the association between RAD symptoms and prosocial behavior problems. Support for this effect emerged when prosocial behavior was reported by primary caregivers, but not when these behaviors were reported by the teacher. Therefore, one should be cautious with attributing meaning to this effect as it could have been driven by shared response bias. In this sample of children with special educational needs, caregivers are more at risk to be distressed due to own psychopathology, own negative childhood experiences, current or recent conflicts with partners, or due to the mere concern about their children’s maladaptive development [69, 70]. As a result, they might think more negative about their child’s social behavior leading them to report more RAD symptoms and less prosocial behavior [71]. This could have driven the results and falsely give the impression that SBS knowledge moderates the link between RAD symptoms and prosocial behavior [72]. The fact that no effects were found for teacher reported prosocial behavior further nurtures this concern. Research has repeatedly shown that parent and teacher reports of child social behavior seldomly correspond [73]. This incongruence has been ascribed to differences in roles between parents and teachers [74]. This might even be more problematic when comparing primary caregivers to special education teachers. The latter’s explicit task is to manage child behavioral problems and explosive group dynamics. As a result, caregiver and teacher reports in our study might have relied on very different contexts in which child behavior was observed and evaluated [75]. Regular school teachers have the advantage that they can better compare one child’s behavior to the wide variety of child behaviors they observe in the classroom [75–78]. This might raise the concerns that the primary caregiver report-related effect we found is less valid than the teacher-related lack of an effect. At the same time, special education teachers might have become less sensitive to children’s deficient prosocial behavior due to, for example, habituation [78]. Consequently, the primary caregiver report-related effects we found might be more than a mere shared response bias effect. Supporting the latter interpretation of our finding, it was promising that collapsing both informants’ reports in a score reflecting the variance in prosocial behavior both informants agreed upon replicated the interaction effect. This again seems to argue against the concern that the interaction effect found for primary caregivers reflects mere reporter bias. Further suggesting that our findings might have some validity, is the multimethod nature of our study (interview, pictorial test and questionnaires). It excludes the likelihood that the findings can be attributed to mere shared method variance, which strengthens the relevance of the effects we found.

Overall, the interaction effect suggested that children with more RAD symptoms and less SBS knowledge showed significantly more prosocial behavior problems than children with more RAD symptoms and more SBS knowledge. The interaction effect remained significant after controlling for the effects of age, gender and perceptual intelligence, suggesting the robustness of the effect. Such a buffering effect of secure attachment in at risk children’s symptom severity has been found before in other research. For example, prior research showed that secure attachment attenuates the link between distress in individuals vulnerable to develop psychosis and the severity of the psychotic symptoms [45]. Also, secure attachment has been found to be a protective factor against the severity of social maladaptation symptoms in adolescents with Attention Deficit Hyperactivity Disorder [70]. Thus, it seems reasonable to interpret the current findings as supporting our hypothesis that SBS knowledge acts as an orthogonal dimension on the link between RAD symptoms and prosocial behavior problems.

### Limitations and suggestions for future research

This study had some important limitations, among which the small sample size. Because we wanted to increase the chances of encountering children with RAD symptoms, we chose a clinical sample of children with emotional and behavioral problems, a population that is rather small. However, we showed that this sample size did not suppress the power of the study, so we do not expect the current study’s null effects to become significant in larger samples. Further, in the current study we used a new approach of measuring SBS knowledge, based in a pictorial task. Further research is needed to evaluate the validity of this task. One way to do this is to compare the pictorial task with the original word prompt task to measure SBS knowledge [21]. It might be that the pictorial test needs more finetuning in line with the word prompt task. Moreover, it might be useful to study outcomes of this pictorial task in different populations of children to check its generalizability. The more knowledge about the validity of the pictorial task, the more precise we can interpret the results of the current study. Additionally, we controlled for perceptual intelligence, but not for other psychological problems or diagnoses that could have affected SBS knowledge or prosocial behavior. For example, Wright and Mccathren suggest that diagnoses like Autism Spectrum Disorder could reduce prosocial behavior [79], so it is worthwhile to take this into account in future research. However, in the current data, we did not have access to validly assessed Autism Spectrum Disorder diagnoses of the children. Moreover, another suggestion for future research is to include a measure of emotion regulation. As explained above, the interaction we found between RAD symptoms and SBS knowledge in the prediction of prosocial behavior was expected because all factors relate to emotion (dys) regulation [17, 18]. Therefore, future research that includes a measure of emotion regulation can further contribute to our understanding of the currently identified pattern of results. A last limitation of this study is that we had no observational measures of RAD symptoms and prosocial behavior. It might be that repeating the current study with such observational measures could help better understand the interplay between RAD symptoms, SBS knowledge, and prosocial behavior.

## Conclusion

The current study was the first to investigate the role of attachment representation (i.c., SBS knowledge) in the association between RAD symptoms and prosocial behavior problems. Results suggested that attachment representations might indeed be important to understand this association, but that they act as a moderator and not as a mediator in the RAD symptoms-prosocial behavior problems association. RAD symptoms and SBS knowledge were not related in this study, which is important in light of the growing discussion on the relevance of attachment theory to understand RAD symptoms [24, 27, 35–37]. Adding to the discussion, the current study calls for careful reasoning to a literature that is characterized by a growing demand to discard the connection between attachment theory and the Attachment Disorders diagnoses [80, 81]. Although our findings seem to fuel such arguments, results also suggested that SBS knowledge played specifically in children with more RAD symptoms a role in the severity of the prosocial behavior problems they displayed. This suggests that merely ignoring attachment theory while thinking about (the treatment of) children with RAD symptoms might be equivalent to throwing the baby out with the bathwater. Although it is not unlikely that the RAD symptoms might more directly reflect children’s behavioral dysregulation [36] or exposure to trauma [38] and less directly reflect children’s lack of trust in the availability of their attachment figures, the moderating effect of SBS knowledge on symptom severity we found in children with RAD symptoms in the current study suggests that restoring/stimulating SBS knowledge could be a relevant component in the treatment of children with RAD symptoms. It is unlikely that this will completely solve the RAD symptoms, but it is likely that RAD symptoms children who can turn more easily to their primary caregivers for support will show a reduction in the severity of their symptoms. Future research should investigate whether these children could also benefit most from therapies that try to repair ruptures or that try to stimulate SBS development. Promising avenues to reach those goals are Attachment-Based Family Therapy [82] or Video-feedback Intervention to promote Positive Parenting and Sensitive Discipline (VIPP-SD) [83].

## Supplementary Information

**Additional file 1: Figure S1.** a. Mediation effect of SBS knowledge in the link between RAD symptoms and prosocial behavior reported by primary caregiver, controlled for gender, age and perceptual intelligence. b. Mediation effect of SBS knowledge in the link between RAD symptoms and prosocial behavior reported by teacher, controlled for gender, age and perceptual intelligence. c. Mediation effect of SBS knowledge in the link between RAD symptoms and prosocial behavior as a mean standardized score of both primary caregiver and teacher reports (compound score), controlled for gender, age and perceptual intelligence.

## Data Availability

The datasets generated and/or analyzed during the current study are not publicly available due to privacy reasons and participant confidentiality but are available from the corresponding author on reasonable request. The data is safely stored at KU Leuven, both digital and on paper. All data were analyzed with IBM SPSS.

## Abbreviations

RAD symptoms: Reactive Attachment Disorder symptoms
SBS: Secure Base Script

## Footnotes

1 Nevertheless, we repeated the analyses for accuracy alone. Supporting our concern that this would be a suboptimal approach to the data, all effects we found with the combination score disappeared (B’s ranged between −.000 and 1.989, *p*-values ranged between .54 and .93).

2 All analyses were also repeated with a factor score of primary caregiver and teacher reports of prosocial behavior as well. Both reports loaded .77 on the latent prosocial score. Only one eigenvalues > 1, 1.18, explaining 59.22% All analyses yielded similar results as the analyses with the mean standardized scores that were reported.
