# Supplementary material for: Reactive attachment disorder symptoms and prosocial behavior in middle childhood: the role of Secure Base Script knowledge
Source: BMC Psychiatry. 2020 Nov 4;20:524. doi: 10.1186/s12888-020-02931-3 (PMC7641862; doi:10.1186/s12888-020-02931-3)
Supplement: Supplementary file 1 — Additional file 1: Figure S1. a. Mediation effect of SBS knowledge in the link between RAD symptoms and prosocial behavior reported by primary caregiver, controlled for gender, age and perceptual intelligence. b. Mediation effect of SBS knowledge in the link between RAD symptoms and prosocial behavior reported by teacher, controlled for gender, age and perceptual intelligence. c. Mediation effect of SBS knowledge in the link between RAD symptoms and prosocial behavior as a mean standardized score of both primary caregiver and teacher reports (compound score), controlled for gender, age and perceptual intelligence. [file 12888_2020_2931_MOESM1_ESM.docx]

RAD symptoms

Prosocial behavior

by primary caregiver

SBS knowledge

β = -3.50, SE = 1.94

Direct effect, β = -2.25*, SE = 0.89

β = 0.06, SE = 0.05

Indirect effect, β = -0.22, 90% CI [-0.72, 0.15]

*Figure S1a*. Mediation effect of SBS knowledge in the link between RAD symptoms and prosocial behavior reported by primary caregiver, controlled for gender, age and perceptual intelligence.

RAD symptoms

Prosocial behavior

by teacher

SBS knowledge

β = -3.27, SE = 2.01

Direct effect, β = -0.97, SE = 0.95

β = 0.06, SE = 0.05

Indirect effect, β = -0.18, 90% CI [-0.71, 0.19]

*Figure S1b*. Mediation effect of SBS knowledge in the link between RAD symptoms and prosocial behavior reported by teacher, controlled for gender, age and perceptual intelligence.

RAD symptoms

Prosocial behavior

compound score

SBS knowledge

β = -3.50, SE = 1.94

Direct effect, β = -0.66*, SE = 0.27

β = 0.02, SE = 0.02

Indirect effect, β = -0.07, 90% CI [-0.23, 0.04]

*Figure S1c*. Mediation effect of SBS knowledge in the link between RAD symptoms and prosocial behavior as a mean standardized score of both primary caregiver and teacher reports (compound score), controlled for gender, age and perceptual intelligence.
